# Irritable Brain Caused by Irritable Bowel? A Nationwide Analysis for Irritable Bowel Syndrome and Risk of Bipolar Disorder

**DOI:** 10.1371/journal.pone.0118209

**Published:** 2015-03-13

**Authors:** Chia-Jen Liu, Li-Yu Hu, Chiu-Mei Yeh, Yu-Wen Hu, Pan-Ming Chen, Tzeng-Ji Chen, Ti Lu

**Affiliations:** 1 Department of Psychiatry, Kaohsiung Veterans General Veterans Hospital, Kaohsiung, Taiwan; 2 Division of Hematology and Oncology, Department of Medicine, Taipei Veterans General Hospital, Taipei, Taiwan; 3 Institute of Public Health & School of Medicine, National Yang-Ming University, Taipei, Taiwan; 4 Department of Family Medicine, Taipei Veterans General Hospital, Taipei, Taiwan; 5 Cancer Center, Taipei Veterans General Hospital, Taipei, Taiwan; 6 Department of Psychiatry, Yuanshan & Suao Branch, Taipei Veterans General Hospital, Yilan, Taiwan; 7 School of Medicine, National Yang-Ming University, Taipei, Taiwan; University Hospital Llandough, UNITED KINGDOM

## Abstract

**Objective:**

We explored the association between IBS and the development of bipolar disorder, and the risk factors for bipolar disorders in patients with IBS.

**Methods:**

We identified patients who were newly diagnosed with IBS between 2000 and 2010 in the Taiwan National Health Insurance Research Database. We also identified a comparison matched cohort without IBS. The occurrence of new-onset bipolar disorder was evaluated in both cohorts.

**Results:**

The IBS cohort consisted of 30,796 patients and the comparison cohort consisted of 30,796 matched patients without IBS. The incidence of bipolar disorder (incidence rate ratio, 2.63, 95% confidence interval (CI) 2.10–3.31, *P* < .001) was higher in the IBS patients than in the matched cohort. Multivariate matched regression models indicated that autoimmune diseases (HR 1.52, 95% CI 1.07–2.17, *P* = .020), and asthma (HR 1.45, 95% CI 1.08–1.95, *P* = .013) were independent risk factors for the development of bipolar disorder in the IBS patients.

**Conclusion:**

IBS may increase the risk of developing subsequent bipolar disorder. Additional prospective studies are required to confirm these findings.

## Introduction

Irritable bowel syndrome (IBS) is a common debilitating gastrointestinal disorder. Depending on the diagnostic criteria employed (Manning, Rome I, Rome II, Rome III), IBS affects around 11% of the population globally [1]. However, the epidemiology of IBS is not well known due to the fact that the studies have used different diagnostic criteria and variability of the studied populations has made it harder to adequately determine the real prevalence of IBS worldwide. Few epidemiological studies have estimated the prevalence of IBS in Asian countries. Prevalence estimates for IBS in China are lower than those in Western populations, ranging from 4% to 6% [2,3]. IBS seems to be more common in the ages between 20 and 40. In most Western nations, IBS is reported more frequently by women than men, but the female predominance reported in the West has not always been reproduced in other countries [4]. This functional gastrointestinal disorder is characterized by episodic exacerbations of symptoms such as abdominal pain, bloating, and altered bowel habits, including diarrhea and/or constipation [5].

Bipolar disorder, also known as manic-depressive disorder, is characterized by transitions between both elevated or irritable mood and depression. Bipolar disorder occurs about as frequently in women and as in men and the usual age at onset is from the teens to 30 years. Depression is a core symptom of bipolar disorder and a number of studies have described depressive symptoms are more common in bipolar disorder than manic symptoms [6]. The National Comorbidity Survey in the United States found that lifetime prevalence of bipolar disorder has generally been estimated at 2% [7]. In Taiwan, the prevalence of bipolar disorder increased from 0.06% in 1996 to 0.4% in 2003. According to the Taiwan National Health Insurance Research Database, there are many patients who were not recognized and treated [8] and patients with bipolar disorder are often misdiagnosed as depression or anxiety disorders, especially on initial presentation.

Investigators have shown increasing interest in the association between psychiatry and gastroenterology, particularly the possibility of psychiatric intervention in patients with functional gastrointestinal disorders, such as IBS. The association between IBS and psychiatric illness has been well described, with the symptoms of psychiatric illness often suggestive of anxiety and depressive disorders [9–11]. These studies have provided evidence to suggest that IBS is a disorder with a psychosomatic aspect. It is widely accepted that chronic inflammation and brain-gut axis dysfunction can explain the association between IBS and psychiatric disorders; however, the underlying pathophysiology is not well understood [5].

During the past decade, inflammation has been revisited as a vital etiologic factor of bipolar disorder [12]. In addition, studies have indicated that numerous cytokines circulating in the plasma might impair blood-brain barrier function [13], suggesting that peripheral inflammation is associated with the upregulation of central nervous system inflammation [14]. However, the pathophysiology of bipolar disorder, in which inflammatory components play crucial roles [15], have not been adequately described.

Studies have indicated that patients with bipolar disorders might be associated with a greater number of medical conditions, including IBS [16,17]. In 2003, Crane et al. conducted a time-series analysis to observe the relationship between the mood changes and IBS symptom severity in a patient with bipolar disorder [18]. Although they did not ignore the possibility that disturbed mood may be a consequence of the experience of IBS, their hypothesis was that disturbed mood may be causal in the relationship. Therefore, the association between IBS and subsequent risk for bipolar disorder has not been well established. In our study, we hypothesize that a history of IBS might increase the risk of developing bipolar disorder.

To evaluate this hypothesis, we conducted a nationwide population-based analysis to investigate the incidence of bipolar disorder in patients with IBS in Taiwan. We also examined the risk factors for bipolar disorder in the patients with IBS.

## Patients and Methods

### Data source

The Taiwan National Health Insurance (NHI) program, which was initiated in 1995, is a mandatory universal health insurance program offering comprehensive medical care coverage to all residents of Taiwan, with a coverage rate of over 98% of the population. Almost 99% of hospitals and clinics in Taiwan are contracted to the NHI program [19]. The program provides coverage for outpatient, inpatient, emergency, and traditional Chinese medicine services, as well as prescription drugs. Multiple NHI databases, including the NHI enrollment files, claims data, and a prescription drug registry, are managed and publicly released by the National Health Research Institutes (NHRI) of Taiwan. The Institutional Review Board of Taipei Veterans General Hospital approved this study (2013–03–035AC). Written consent from the study patients was not obtained, because the NHI dataset consists of de-identified secondary data for research purposes and the Institutional Review Board of Taipei Veterans General Hospital issued a formal written waiver for the need for consent. Detailed information on data requests is provided on the NHRI Web site (http://nhird.nhri.org.tw).

### Study design and participants

A retrospective cohort study was conducted using patients who were newly diagnosed with IBS between January 1, 2000, and December 31, 2010. Patients with IBS were identified in the Taiwan National Health Insurance Research Database (NHIRD) based on the International Classification of Diseases, 9th revision, Clinical Modification (ICD-9-CM) code 564.1 and the criteria had been used in similar studies [20,21]. To increase the validity of IBS diagnosis, we excluded data on those who had fewer than 3 visits, and their diagnosis was altered within 3 months following the index date [22]. We defined the first IBS diagnosis in the database as the index date, and we excluded patients with IBS before the year 2000 to ensure that our study population had no IBS diagnosis prior to enrolment in this study. In addition, the study recruited only patients aged 20 years or older at the time of IBS diagnosis who had no previous history of bipolar disorder on the date of IBS diagnosis.

Bipolar disorder was defined by compatible ICD-9-CM codes (296.0X-296.1X, 296.4X-296.8X, or 296.86 [23,24]) between January 1, 2000 and December 31, 2010. To enhance the reliability of the bipolar disorder diagnoses, cases of diagnosed bipolar disorder were included only when the diagnostic process or assessment was performed by a qualified psychiatrist in Taiwan. Moreover, we collected information on the use of psychotropic agents approved by the Food and Drug Administration of Taiwan for treating one (or more) phases of bipolar disorder, including acute mania/mixed episodes, bipolar depression and bipolar maintenance. In addition to mood stabilizers, atypical antipsychotics (e.g., aripiprazole, olanzapine, quetiapine, risperidone, and ziprasidone) were included. These drugs were classified according to the World Health Organization Anatomical Therapeutic Chemical Classification System. Only patients who were prescribed these drugs for at least one month were included in our study.

For each patient with IBS in the NHIRD, one patient without an IBS diagnosis during the study period were randomly matched for age, sex, comorbidities and enrollment date. The IBS and comparison cohorts were followed up until the development of bipolar disorders, death, or the end of the study period.

### Statistical analysis

A diagnosis of bipolar disorder served as the primary dependent variable. The bipolar disorder incidence rate (per 1000 person-years) and the incidence rate ratio (IRR) were calculated in each study group. The groups were compared using the chi square test for categorical variables. The Kaplan-Meier method was used to estimate the cumulative incidence of bipolar disorder, and a Cox proportional hazards model was used to identify the risk factors for bipolar disorder in the patients with IBS. The qualifying criterion for inclusion in the multivariate analysis was a result in the univariate analysis with a *P* value <. 1. The Perl programming language (version 5.12.2) was used to extract the data from the databases. Microsoft SQL Server 2005 (Microsoft Corp., Redmond, WA, USA) was used for data linkage, processing, and sampling. The SPSS, version 19.0 for Windows (IBM, Armonk, NY, USA), and SAS, version 9.2 (SAS Institute, Cary, NC, USA), computer software programs were used to perform all statistical analyses. A *P* value <. 05 was considered statistically significant.

## Results

### Participant characteristics

Table 1 displays the demographic and comorbidity data for the IBS patients and matched cohort. The median age of all patients was 50 years (interquartile range, 40–66 years). When we stratified our study population to 3 groups (20–39, 40–59 and ≥60 years), the majority of the patients were aged 40–59 years (40%) and the median age of the 20–39 and 40–59 groups was 40.2 years. Hypertension, dyslipidemia, chronic obstructive pulmonary disease, and diabetes mellitus were the most common comorbidities. The baseline comorbidity data of the 2 study groups showed nonsignificant differences.

**Table 1:** Baseline patient characteristics of patients with and without irritable bowel syndrome.

Demographic data	Patients with irritable bowel syndrome		Matched cohort		*P* value
	*n* = 30,796		*n* = 30,796		
	*n*	%	*n*	%	
**Age (years)** ^a^	50 (40–66)		50 (40–66)		
**Distribution of age**					
20–39	7,698	25.0	7,698	25.0	1.000
40–59	12,329	40.0	12,329	40.0	1.000
≥60	10,769	35.0	10,769	35.0	1.000
**Sex**					
Male	14,536	47.2	14,536	47.2	1.000
Female	16,260	52.8	16,260	52.8	
**Comorbidities**					
Autoimmune diseases	2,934	9.5	2,922	9.5	0.869
Chronic kidney disease	4,074	13.2	4,074	13.2	1.000
Cerebrovascular diseases	4,784	15.5	4,783	15.5	0.991
Diabetes mellitus	7,059	22.9	7,056	22.9	0.977
Hypertension	11,426	37.1	11,423	37.1	0.980
Asthma	5,221	17.0	5,211	16.9	0.915
COPD*	8,477	27.5	8,474	27.5	0.978
Malignancies	1,150	3.7	1,143	3.7	0.882
Cirrhosis	1,001	3.3	984	3.2	0.698
Dyslipidemia	9,385	30.5	9,376	30.4	0.937
Coronary artery disease	553	1.8	530	1.7	0.481
**Follow-up years (median)**	5.66 (3.17–8.34)		5.62 (3.16–8.30)		0.320

^a^ Median (Interquartile range)

* COPD, chronic obstructive pulmonary disease

### Incidence of bipolar disorder

**Fig 1:**
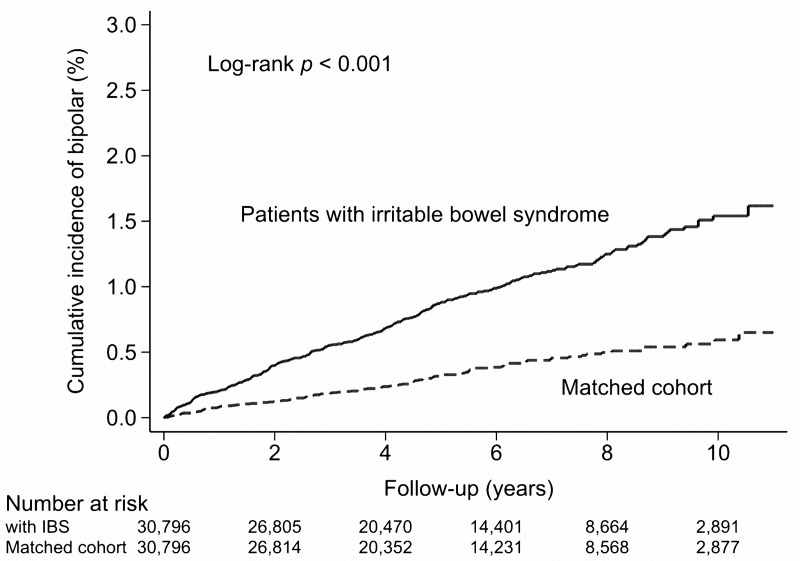
Cumulative incidence of subsequent bipolar disorders in patients with and without Irritable bowel syndrome.

Fig. 1 displays the cumulative incidences of bipolar disorders in the study patients. As shown in Table 2, the risk of developing bipolar disorder was significantly higher in the patients with IBS than in the matched patients (IRR, 2.63; 95% confidence interval [CI] 2.10–3.31; *P* <. 001). When we stratified the patients according to age and sex, the IBS cohort exhibited a similarly increased risk of developing bipolar disorder compared to the matched cohort. When we stratified the patients according to the duration of follow-up, we observed the risk of developing bipolar disorder remained higher in the IBS cohort with longer durations of follow-up (1–5 y and ≥5 y). Overall, the incidence of bipolar disorder after a diagnosis of IBS was 1.6 per 1000 person-years.

**Table 2:** Incidence of bipolar disorder in patients with and without irritable bowel syndrome.

	Patients with irritable bowel syndrome		Matched cohort		IRR (95% CI)	*P* value
	Bipolar No.	Per 1,000 person-year	Bipolar No.	Per 1,000 person-year		
**Total**	288	1.6	109	0.6	2.63 (2.10–3.31)	<0.001
**Age**						
20–39	84	1.8	32	0.7	2.62 (1.72–4.07)	<0.001
40–59	124	1.7	52	0.7	2.39 (1.72–3.37)	<0.001
≥60	80	1.4	25	0.4	3.16 (1.99–5.16)	<0.001
**Sex**						
Male	116	1.4	39	0.5	2.95 (2.03–4.35)	<0.001
Female	172	1.8	70	0.7	2.46 (1.85–3.29)	<0.001
**Follow-up**						
0–0.5 year	39	2.5	11	0.7	3.55 (1.78–7.68)	<0.001
0.5–1 year	24	1.6	15	1.0	1.60 (0.81–3.28)	0.154
1–5 year	160	1.7	55	0.6	2.90 (2.12–4.02)	<0.001
≥ 5 year (5–11 year)	65	1.3	28	0.5	2.30 (1.45–3.72)	<0.001

IRR, incidence rate ratio; CI, indicates confidence interval

### Risks factors for bipolar disorders in patients with IBS

In the univariate and multivariate analyses, autoimmune diseases and asthma were independent risk factors for the development of bipolar disorder in the IBS patients (Table 3).

**Table 3:** Analyses of risk factors for subsequent bipolar disorder in patients with irritable bowel syndrome.

Predictive variables	Univariate analysis		Multivariate analysis	
	HR (95% CI)	*P* value	HR (95% CI)	*P* value
**Age**	0.99 (0.98–1.00)	0.006	0.99 (0.98–1.00)	<0.001
**Sex** (female)	1.30 (1.03–1.64)	0.030	0.80 (0.63–1.01)	0.059
**Comorbidities**				
Autoimmune diseases	1.57 (1.10–2.22)	0.012	1.58 (1.11–2.25)	0.012
Chronic kidney disease	1.07 (0.75–1.52)	0.723		
Cerebrovascular diseases	1.27 (0.93–1.73)	0.139		
Diabetes mellitus	1.14 (0.87–1.51)	0.342		
Hypertension	1.17 (0.92–1.49)	0.198		
Asthma	1.42 (1.06–1.90)	0.019	1.50 (1.12–2.02)	0.007
COPD	1.07 (0.82–1.39)	0.609		
Malignancies	1.09 (0.56–2.12)	0.799		
Cirrhosis	1.26 (0.67–2.37)	0.468		
Dyslipidemia	1.17 (0.91–1.50)	0.223		
Coronary artery disease	1.36 (0.61–3.06)	0.452		

COPD, chronic obstructive pulmonary disease

## Discussion

Our study is the first population-based analysis to examine IBS might increase the risk of developing bipolar disorder by using matched cohorts and a long-term (11 years) follow-up. Our major finding was a higher incidence of bipolar disorder in the patients with IBS compared with the matched cohort. In addition, we determined that the comorbid conditions of autoimmune diseases and asthma were associated with a higher risk of developing bipolar disorder among the IBS patients.

Hao *et al*. conducted a small-scale study to assess the prevalence of psychiatric comorbidities in patients with IBS, observing that mood disorders (which include depressive and bipolar disorders) are the most common psychiatric comorbidities (22.9%) [10]. However, the authors did not perform a subanalysis of the prevalence of bipolar disorder. In our nationwide study, focusing on bipolar disorder, the incidence of subsequent bipolar disorders in the IBS patients was 1% during the 11-year follow-up.

Two possible mechanisms could underlie our observation on increased risk of developing bipolar disorders in the patients with IBS. First, the development of bipolar disorder after IBS might be caused by an inflammatory process activated by IBS [5]. The chronic peripheral inflammatory process activated by IBS might increase the risk of the upregulation of CNS inflammation [14]. Besides, previous studies have indicated that even mild inflammation in the periphery might be a precipitating factor for the CNS inflammation, which is associated with the development of bipolar disorder [25,26]. Moreover, the dysregulated brain inflammatory processes caused by peripheral inflammation might turn to trigger and propagate systemic atherosclerosis, hypertension, diabetes, obesity, and hyperthyroidism [27–30], leading to a vicious circle effect. Animal models have also demonstrated that peripheral cytokines reach the brain through various mechanisms, including a leaky brain barrier, activation of endothelial cells, and binding to cytokine receptors, and that these cytokines interact with pathophysiologic domains relevant to bipolar disorder [31]. Second, we propose that the risk factors for IBS and bipolar disorder, such as stresses, are the same [5]. Stressful psychosocial factors can induce IBS, and can also increase the likelihood of developing bipolar disorder in patients with genetic vulnerability [32,33].

After stratification according to the duration of follow-up, the incidence of bipolar disorder was higher in the IBS patients than in the matched cohort within the first 6 months of a diagnosis of IBS, with an IRR of 3.55. One possible explanation for this result is surveillance bias [34]. However, after excluding the first year of observation, no compensatory decreased risk existed among patients with IBS in the follow-up period. This indicates a more direct link between IBS and the subsequent bipolar disorder.

Our analyses further indicated that autoimmune disease is an independent risk factor for the development of bipolar disorders in IBS patients. Rege *et al*. reported that immune dysregulation and autoimmunity are associated with the pathophysiology of bipolar disorders [35]. In addition, the IBS patients with a history of asthma also exhibited a higher risk of developing bipolar disorder. Previous studies have indicated that asthma is a common comorbidity in patients with bipolar disorder, and might be associated with a significantly increased likelihood of developing bipolar disorder [36]. Allergy-related immunological dysregulation might explain the comorbid association between asthma and bipolar disorder [37,38]. These reports partially supported our hypothesis. Moreover, in our study, after stratification according to the number of these immune-related diseases, there was higher risk of developing bipolar disorder among the patients with IBS if there were more than one immune-related disease. Therefore, the association between IBS and bipolar disorder may be explained by the inflammation process.

Our study is the first retrospective cohort studies to evaluate the association between the IBS and the subsequent bipolar disorder. The nationwide matched-cohort study design using a cohort of the IBS patients and adequate controls for comorbidities constitute the strengths of our study. However, it has several limitations inherent to the use of claims databases that should also be considered. First, the *P* value depends essentially on the effect size and the sample size. Therefore, study using large sample size such as nationwide database may emphasize the statistical significant result rather than the importance of the effect size. Second, the reliability and validity of the diagnoses of IBS and bipolar disorder remain a major concern. To ensure the validity of IBS diagnosis, the identification of IBS patients was based not only on the ICD-9-CM codes. We attempted to increase the validity of IBS diagnosis by excluding those who had fewer than 3 visits or their IBS diagnosis was altered within 3 months of the index date, which was similar to the IBS definition used in other studies using the NHIRD source in Taiwan [22]. For improving the validity of bipolar disorder diagnosis, in addition to the ICD-9-CM code [23], medication use for bipolar disorder such as mood stabilizers and/or atypical antipsychotics were further analyzed in our study [29]. However, because of the NHIRD data protection policy, it was impossible to validate the subjects’ data in the medical records to confirm the diagnoses of IBS and bipolar disorder. Third, it is worth mentioning that since our study is based on an observational design rather than experimental one, the association between the IBS and the subsequent newly diagnosed bipolar disorder development in IBS patients does not lead us to infer a causal relationship, especially for the study which retrieved national database and the result revealed a relatively low HR. Fourth, several demographic variables were unavailable, such as family history of bipolar disorder and IBS, environmental exposures, diet, and lifestyle [39–41]. Fifth, data on the subtypes of IBS and the severity of IBS were limited [42]. Finally, according to epidemiological analyses, although IBS is common worldwide, the similarities and differences between geographical regions and cultural groups remain unclear [43]. In our study, we were unable to exclude the possibility that the population with IBS in the NHIRD might be composed of people of different races and cultures.

In conclusion, our study results indicate that IBS might increase the risk of subsequent bipolar disorder. Our results also suggest that comorbidities including autoimmune diseases and asthma may be risk factors for developing bipolar disorder following a diagnosis of IBS. Further prospective clinical studies on the association between IBS and bipolar disorder are warranted.
